# Changes in Micronutrient Intake and Status, Diet Quality and Glucose Tolerance from Preconception to the Second Trimester of Pregnancy

**DOI:** 10.3390/nu11020460

**Published:** 2019-02-22

**Authors:** Moniek Looman, Anouk Geelen, Rahul A. K. Samlal, Rik Heijligenberg, Jacqueline M. T. Klein Gunnewiek, Michiel G. J. Balvers, Lia D. E. Wijnberger, Elske M. Brouwer-Brolsma, Edith J. M. Feskens

**Affiliations:** 1 Division of Human Nutrition, Wageningen University, P.O. Box 17, 6700 AA Wageningen, The Netherlands; anouk.geelen@wur.nl (A.G.); elske.brouwer-brolsma@wur.nl (E.M.B.-B.); edith.feskens@wur.nl (E.J.M.F.); 2 Department of Gynaecology and Obstetrics, Hospital Gelderse Vallei Ede, P.O. Box 9025, 6710 HN Ede, The Netherlands; SamlalR@zgv.nl; 3 Department of Internal Medicine, Hospital Gelderse Vallei Ede, P.O. Box 9025, 6710 HN Ede, The Netherlands; HeijligenbergR@zgv.nl; 4 Clinical Chemistry and Haematology Laboratory, Hospital Gelderse Vallei Ede, P.O. Box 9025, 6710 HN Ede, The Netherlands; KleinGunnewiekJ@zgv.nl (J.M.T.K.G.); BalversM@zgv.nl (M.G.J.B.); 5 Department of Obstetrics and Gynaecology, Rijnstate Hospital, P.O. Box 9555, 6800 TA Arnhem, The Netherlands; LWijnberger@rijnstate.nl

**Keywords:** diet quality, gestational diabetes, micronutrients, pregnancy, status markers, glucose homeostasis, folate, vitamin B6, vitamin B12, vitamin D, iron

## Abstract

Data on changes in dietary intake and related blood parameters throughout pregnancy are scarce; moreover, few studies have examined their association with glucose homeostasis. Therefore, we monitored intake of folate, vitamin B6, vitamin B12, vitamin D and iron, their status markers, and diet quality from preconception to the second trimester of pregnancy, and we examined whether these dietary factors were associated with glucose homeostasis during pregnancy. We included 105 women aged 18–40 years with a desire to get pregnancy or who were already <24 weeks pregnant. Women at increased gestational diabetes (GDM) risk were oversampled. Measurements were scheduled at preconception (*n* = 67), and 12 (*n* =53) and 24 weeks of pregnancy (*n* =66), including a fasting venipuncture, 75-grams oral glucose tolerance test, and completion of a validated food frequency questionnaire. Changes in micronutrient intake and status, and associations between dietary factors and glucose homeostasis, were examined using adjusted repeated measures mixed models. Micronutrient intake of folate, vitamin B6 and vitamin D and related status markers significantly changed throughout pregnancy, which was predominantly due to changes in the intake of supplements. Micronutrient intake or status levels were not associated with glucose homeostasis, except for iron intake (FE µg/day) with fasting glucose (β = −0.069 mmol/L, *p* = 0.013) and HbA1c (β = −0.4843 mmol, *p* = 0.002). Diet quality was inversely associated with fasting glucose (β = −0.006 mmol/L for each DHD15-index point, *p* = 0.017). It was shown that micronutrient intakes and their status markers significantly changed during pregnancy. Only iron intake and diet quality were inversely associated with glucose homeostasis.

## 1. Introduction

An adequate dietary intake and nutrient status during pregnancy is required to ensure optimal fetal development [1]. However, nutrient deficiencies are common during pregnancy, even in high-income countries, due to the increasing consumption of high-fat, high-sugar diets with low nutrient densities [2]. A recent review indicated that pregnant women are particularly at risk of inadequate intake of iron, folate and vitamin D [3]. As micronutrients are involved in the formation of new cells and tissues, enzyme activity, signal transduction and transcription pathways, and combating oxidative stress, micronutrient inadequacies may be adversely associated with both maternal and fetal metabolic processes [4,5]. 

Gestational diabetes mellitus (GDM) is one of the most common metabolic pregnancy complications. GDM is defined as hyperglycaemia with onset or first detection during pregnancy; the condition affects approximately 7% of pregnancies [6,7]. Several large prospective cohort studies have suggested associations of preconception dietary intake with GDM diagnosis [8,9,10]. In agreement with this, several randomized, controlled trials showed significant effects of dietary counselling during pregnancy on GDM prevention [11,12]. However, studies examining associations between nutritional components and GDM reporting null associations have been published as well [13]. Multiple studies have investigated the role of specific micronutrients in relation to GDM. Higher iron intake and low vitamin D intake have been associated with a higher risk of GDM [14,15,16]. Studies that focused on nutrient status observed associations of higher status levels of vitamin B12 and vitamin D [17,18,19,20] and lower status levels of folate and iron [16,19] with a lower GDM risk. However, it should be noted that some of these studies had a cross-sectional or retrospective design, and did not account for changes in dietary intake or nutrient status throughout pregnancy. 

Ideally, studies investigating the potential impact of micronutrients on health outcomes should measure both micronutrient intake and status, i.e., providing insight in both actual intakes as well as nutrient status. In case of pregnancy, the impact of maternal haemodynamic, cardiovascular, renal and gastrointestinal adaptations on micronutrient levels needs to be considered as well. To date, only few studies have described micronutrient status levels throughout pregnancy [21,22,23,24,25], and therefore, very little is known about the optimal micronutrient status for pregnant women. In addition, to our knowledge, none of these previous studies have examined the impact of dietary intake and supplement use during pregnancy. 

Given this lack of data on the optimal nutrient levels during pregnancy, the aim of this study was (1) to describe changes in selected micronutrients intake, i.e., folate, vitamin B6, vitamin B12, vitamin D and iron, their status markers and overall diet quality from preconception to the second trimester of pregnancy, and (2) to examine association of micronutrients and overall diet quality with glucose tolerance during pregnancy in a sample of healthy women at increased risk of GDM with a singleton pregnancy.

## 2. Materials and Methods

### 2.1. Study Design and Participants

This study was performed using data from the GLIMP2 study, a small prospective cohort study aiming to assess the role of diet, nutrient status and other risk factors in the development of GDM. Women with a wish to get pregnant within one year or those less than 24 weeks pregnant were recruited between June 2015 and May 2017 at the Department of Gynaecology and Department of Internal Medicine at three non-university hospitals in the eastern part of the Netherlands: Gelderse Vallei Hospital (Ede), Rijnstate (Arnhem), and Slingeland (Doetinchem). Women with a higher risk of developing GDM (i.e., previous pregnancy with GDM or macrosomic infant or overweight/obese) were intentionally oversampled. The main inclusion criteria were age between 18 and 40 years, willing to get pregnant within one year or less than 24 weeks pregnant at time of recruitment, and showing competence to make their own decisions. Women were excluded when they were not able to read and speak Dutch. Measurements were scheduled before pregnancy (T0), at 12 weeks of gestation (T1), and at 24 weeks of gestation (T2). Women started at either T0, T1 or T2, depending on the recruitment date. All participants filled out a baseline questionnaire at the start of the study. At each time point, participants visited one of the research centers. Measurements included anthropometrics, a fasting venipuncture followed by a 75-grams oral glucose tolerance test (OGTT) including a venipuncture 2 h after the glucose load, filling out a food frequency questionnaire (FFQ) and questionnaires on lifestyle, health and pregnancy-related factors. The Medical Ethics Committee of Wageningen University & Research approved the study. All women gave their written informed consent before the start of the study.

### 2.2. Analysis Sample

**Figure 1:**
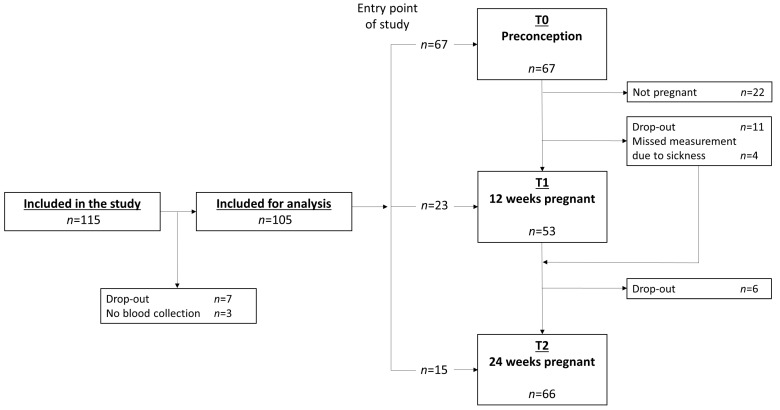
Flowchart of the GLIMP2 study.

In total, 115 women were included in the study (Figure 1). Seven women dropped out before completing any of the measurements, and 3 women were excluded for the current analyses because of missing data on blood measurements, leaving a total of 105 women for the current analyses. In total, 67 women completed a measurement at T0, 53 women completed a measurement at T1 and 66 women completed a T2 measurement. Fifty-seven women completed at least two measurements and 24 women completed three measurements. Thirty-five women completed a T0 measurement and at least one measurement during pregnancy (T1/T2). Eleven women dropped out after T0. The main reasons for dropping out were lack of time, and the burden of the OGTT. Women who did not get pregnant after T0 measurements (*n* = 22) did not differ significantly regarding age, BMI, ethnicity, education level and smoking status from those who did become pregnant within 12 months after T0 measurement (*n* = 45) (data not shown). Furthermore, women who completed two or three measurements (*n* = 57) did not differ with respect to age, ethnicity, education level and smoking status from women who completed only one measurement (*n* = 48) (data not shown).

### 2.3. Dietary Assessment 

#### 2.3.1. Food Frequency Questionnaire (FFQ)

A semi-quantitative 173- item FFQ was used to assess usual dietary intake of the previous month. The FFQ was designed and validated to estimate habitual dietary intake of energy, macronutrients, fiber and B-vitamins in Dutch adults [26,27,28]. Answer categories for frequency questions ranged between ‘not in this month’ to ‘6–7 days/week’, and portion sizes were estimated using natural portions (bread shapes) and commonly used household measures (e.g. spoon and cup). Average daily nutrient intakes were calculated by multiplying frequency of consumption by portion size and nutrient content per gram using the 2011 Dutch food composition table [29].

#### 2.3.2. Supplement Use

All participants were asked to report whether they used dietary supplements. For each supplement, the frequency, number of tablets or drops, type, and brand were reported. The nutrient content of the supplements was based on the product label information as obtained from the manufacturer. Total micronutrient intake for folate, vitamin B6, B12, D and iron was obtained by summing dietary intake and supplemental intake. To account for differences in bioavailability of natural and synthetic folate, folate intake was expressed as folate equivalents (FE). Total folate intake (FE µg/day) was obtained by summing dietary folate intake (FE µg/day) + 1.7 * supplemental folic acid (µg/day) [30].

#### 2.3.3. Dutch Healthy Diet 2015 (DHD15) Index Score

The Dutch Healthy Diet index 2015 (DHD15-index) was used as measure of diet quality. Dietary intake data from the FFQ was used to calculate the scores. This index was developed based on the Dutch dietary guidelines of 2015 [31]; its design and calculation have been described elsewhere [32]. In brief, the DHD15-index comprises of 15 components on fruits, vegetables, wholegrain products, legumes, nuts, fish, tea, dairy, coffee, fats and oils, red meat, processed meat, sweetened beverages and fruit juices, alcohol and salt. The coffee and sodium component were omitted, as the type of coffee and sodium intake were not assessed. The scores for each component ranged between 0 and 10 points, resulting in a total DHD15-index score ranging from zero to 130 points. Higher scores indicate a higher level of adherence to the Dutch dietary guidelines. 

### 2.4. Biochemical Analysis

Fasting blood samples were obtained by venipuncture in the morning at one of the hospitals followed by a 75-gram OGTT. Blood samples were transported in a cool storage box with a temperature around 7 °C to the Clinical Chemistry and Haematology Laboratory of Gelderse Vallei Hospital (Ede, the Netherlands) and processed within three hours after collection. Plasma fasting and 2-h glucose levels, fasting folate, ferritin and vitamin B12 were analyzed using the Siemens Dimension Vista^®^ System (Siemens Healthcare, The Hague, the Netherlands). For glucose measurement, the hexokinase assay was used. For measurement of folate, ferritin and vitamin B12, a quantitative competitive chemoluminescence assay based on LOCI^®^ technology was used. Plasma ferritin was used as marker for iron status. HbA1c was measured in whole blood with the HA-8180V analyser (Menarini Diagnostics, Florence, Italy). Whole blood vitamin B6 concentrations were determined using a commercially available kit (Chromsystems Instruments & Chemicals GmbH, Gräfelfing, Germany) with isocratic high-performance liquid chromatography (HPLC) and fluorescence detection till March 2017.,After March 2017, a liquid chromatography coupled to tandem mass spectrometry (LC-MS/MS) system consisting of a Waters Acquity UPLC I-Class system, coupled to a Waters Xevo TQ-S micro mass spectrometer (Waters Chromatography B.V., Etten-Leur, the Netherlands) was used. Serum 25(OH)D levels were measured on a HPLC system with UV detector using a commercially-available kit (Chromsystems) till July 2016; after July 2016, a method using the above-described LC-MS/MS system was used.

### 2.5. Covariates

Body weight and height were measured by trained professionals at each visit. Body Mass Index (BMI) was calculated as body weight divided by squared body height (kg/m^2^). Data on maternal age, ethnicity (western/non-western), marital status (married/living together), parity (no/one or more child), educational level (low/intermediate/high), and smoking habits (yes/no), nausea and vomiting during pregnancy were collected using standardized questionnaires. Birth country of the participant and her biological parents was used to determine ethnicity. Highest completed education was classified into three categories; low: primary school, vocational or lower general secondary education, intermediate: higher secondary education or intermediate vocational training and high: higher vocational education or university. Physical activity was assessed using the validated Short Questionnaire to Assess Health-enhancing physical activity (SQUASH) [33]. The duration (minutes per week) and intensity (Total Metabolic Equivalents (MET)) of total and light-, moderate-, and vigorous-intensity physical activities were calculated. Date of blood sampling was used to define a covariate for season (summer/autumn: May–November and winter/spring: December–April) [34]. Alcohol intake was assessed with the FFQ. Participants were categorized into three categories, based on average alcohol intake assessed with the FFQ: abstainers (<0.1 gram of alcohol/day); 0–1 standard glass of alcohol per day (average alcohol intake between 0.1 and 10 gram of alcohol/day) and >1 standard glass of per day (>10 gram of alcohol). A participant was diagnosed with GDM if at least one test value from the OGTT performed at 12 weeks of pregnancy or 24 weeks of pregnancy was abnormal (fasting glucose plasma ≥ 6.1 mmol/L or 2-h plasma glucose ≥ 7.8 mmol/L), according to the diagnostic criteria of the WHO established in 1999 [35].

### 2.6. Statistical Analysis

Participant characteristics of the study population at each time point were reported as median with interquartile range (25th; 75th percentile), or as percentage (%). To test for differences in participant characteristics at T0, T1 and T2, a χ^2^ was used for categorical values and an ANOVA for continuous variables. To describe changes of diet quality, micronutrient status, total intake, dietary intake and supplemental intake of folate, vitamin, B6, B12, D and iron during pregnancy a repeated measures mixed model with time as fixed effect was used, i.e., crude model, as this procedure allowed us to account for missing observations and correlated measurements [36]. The covariance structure resulting in the best model fit based on BIC fit statistic was chosen for each analysis. Adjusted repeated measures mixed models were conducted to assess associations between dietary or supplemental intake with (changes in) micronutrient status while adjusting for covariates. Potential covariates for all models included age, education, ethnicity, parity, smoking, nausea during pregnancy, vomiting during pregnancy, season of blood collection, physical activity, energy intake, alcohol intake, time between measurements, BMI, and intakes and status of the other micronutrients. Adjusted repeated measures mixed models were also conducted to examine associations of micronutrient intakes, micronutrient status and diet quality with markers of glucose homeostasis. Potential confounders included age, education, ethnicity, parity, smoking, season of blood collection, physical activity, energy intake, alcohol intake, time between measurements, history of GDM and BMI. Eventually, all covariates influencing the studied effect estimates >10% were included in the model, i.e., fully adjusted model. A variable taking into account the change in biochemical analyses method for vitamin B6 and vitamin D was created and added to the model in a sensitivity analysis. As this variable was not significant in any of the vitamin B6 or vitamin D analysis and did not influence estimates, it was not included in the final model. Statistical analyses were conducted using SAS Software Version 9.4 (SAS Institute Inc., Cary, NC, USA). A *p*-value of ≤0.05 (two-sided) was considered statistically significant.

## 3. Results

Participants (*n* = 105) were on average 31.9 (25th percentile 29.8; 75th percentile 34.3) years old, and mostly highly educated (61.0%). Five of the 105 women (5.7%) had a non-western ethnicity and the majority of the women (92.4%) were multipara. Characteristics of the study population at each measurement moment are presented in Table 1. Over the course of pregnancy, median BMI increased from 24.4 (22.2;28.4) kg/m^2^ at preconception to 27.0 (24.6;31.4) kg/m^2^ at 24 weeks of pregnancy. Physical activity decreased after women got pregnant and women were on average the least physically active at 12 weeks of pregnancy. Most women (90%) experienced nausea and 32% suffered from vomiting at 12 weeks gestation; these numbers decreased during the second trimester. Energy intake increased during pregnancy from 8583 (6713;9462) kJ at preconception to 9189 (7432;10541) kJ at 24 weeks of gestation. The relative contribution of proteins, carbohydrates and fat consumed remained quite stable throughout pregnancy. The majority of women abstained from alcohol before and during pregnancy, whereas five women reported to consume a small amount of alcohol during their pregnancy. 

### 3.1. Changes in Micronutrient Intake, Micronutrient Status and Diet Quality Throughout Pregnancy 

**Figure 2:**
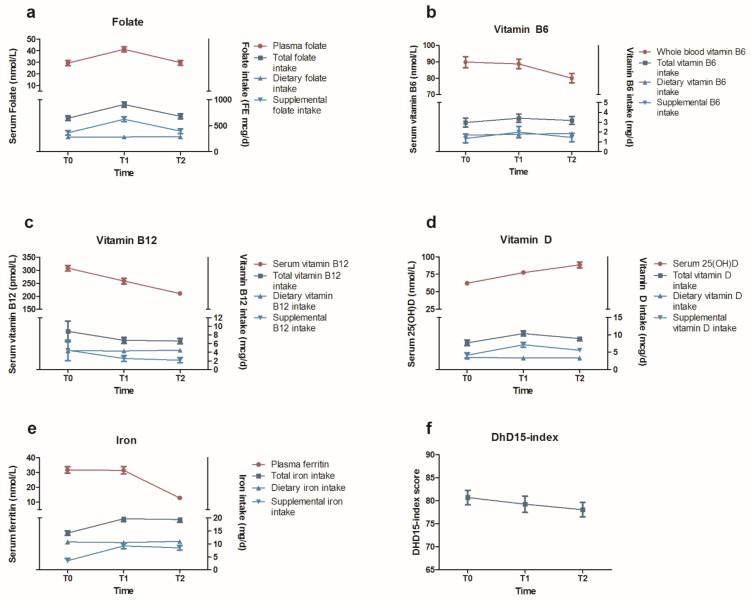
Changes in total, dietary and supplement nutrient intake and status for (**a**) folate, (**b**) vitamin B6, (**c**) vitamin B12, (**d**) vitamin D, (**e**) iron, and (**f**) diet quality as assessed by the DHD15-index. Values are mean (SEM) and presented in Supplementary Data Table S1. T0 is preconception, T1 is 12 weeks pregnant, and T2 is 24 weeks pregnant.

Mean changes in micronutrient intake, micronutrient status and diet quality during pregnancy are presented in Figure 2 and Supplemental Table S1. The estimates of the adjusted repeated mixed model, indicating whether dietary or supplemental intake were associated with (changes in) micronutrient status, are presented in Table 2. The beta-coefficients for time indicate if nutrient status changed significantly over the course of the pregnancy, independent of changes in supplemental or dietary nutrient intake over the course of the pregnancy, whereas the beta-coefficients for supplemental and dietary nutrient intake reflected the association with nutrient status over the course of the pregnancy. 

Total folate intake was highest at T1 (Figure 2a). As dietary folate intake was similar at T0, T1 and T2, this higher total folate intake level at T1 was principally explained by higher supplemental folate intakes at T1. Plasma folate levels significantly increased from 29.3 ± 2.2 nmol/L at T0 to 41.1 ± 2.1 at T1, and were significantly lower at T2 towards levels comparable to T0 (Supplemental Table S1). Adjustment for supplemental folate intake attenuated the association between total folate intake and plasma folate, suggesting that supplemental folate is the main determinant of folate status. In line with this, supplemental folate intake was significantly associated with plasma folate levels (β 0.026 nmol/L for each FE µg, *p* < 0.001) (Table 2). 

Total, dietary and supplemental vitamin B6 intake did not significantly change from T0 to T2 (Figure 2b). Nevertheless, vitamin B6 levels slightly decreased from 89.8 ± 3.4 nmol/L at T0 to 88.7 ± 2.9 nmol/L at T1, and significantly decreased to 80.0 ± 2.8 nmol/L at T2. This significant decrease in vitamin B6 levels from T1 to T2 did not alter after adjustment for vitamin B6 intake. Finally, supplemental vitamin B6 intake was positively associated with vitamin B6 levels (Table 2). 

Total vitamin B12 intake decreased slightly from T0 to T1, and subsequently decreased significantly from T1 to T2, due to a significant decrease in supplemental vitamin B12 intake. Dietary vitamin B12 intake decreased slightly, but not significantly, from T0 to T2 (Figure 2c). Vitamin B12 levels significantly decreased from on average 308.4 ± 10.8 pmol/L at T0 to 258.3 ± 11.0 pmol/L at T1 and further decreased to 210.3 ± 7.6 pmol/L at T2, which were shown to be independent of supplemental and dietary vitamin B12 intake (Table 2). 

Total vitamin D intake significantly increased over the course of the pregnancy, with highest total intake measured at T1 (Figure 2d). As dietary vitamin D intake remained stable throughout pregnancy, these fluctuations in the total vitamin D intake were principally related to supplemental vitamin D intakes. Serum 25(OH)D levels, adjusted for season, significantly increased throughout pregnancy, i.e., from 62.1 ± 3.0 nmol/L at T0 to 77.4 ± 3.1 at T1 and 88.5.0 ± 4.2 nmol/L at T2. Moreover, both the intake of supplemental vitamin D (μg/day) (β = 1.17 nmol/L, *p* < 0.001) and dietary vitamin D (μg/day) (β = 6.66 nmol/L, *p* < 0.001) were significantly associated with 25(OH)D serum levels (Table 2). Adjustment for dietary and supplemental vitamin D intake did not attenuate the observed time-effect, indicating that dietary and supplemental vitamin D intake only partly explain the observed increase in 25(OH)D serum levels during pregnancy.

Total iron intake increased from T0 to T2, which was mainly attributable to a significant increase in supplemental iron intake from T0 to T1 (Figure 2e). Ferritin levels remained stable from T0 to T1 (31.7 ± 2.2 nmol/L and 31.4 ± 2.5 nmol/L, resp.), but significantly decreased to 12.8 ± 1.2 nmol/L at T2. Supplemental and dietary iron intake were not associated with ferritin levels and could not explain the time effect (Table 2).

Diet quality as reflected by the DHD15-index score decreased not significantly from 80.7 ± 1.6 points at preconception to 78.0 ± 1.6 at 24 weeks of pregnancy (*p* = 0.72) (Figure 2f).

### 3.2. Associations of Micronutrient Intake, Micronutrient Status and Diet Quality with Glucose Tolerance During Pregnancy

In total, 12 of the 105 (11.4%) women developed GDM. Of these 12 women, seven were measured at all three time points, and five were measured at T1 and T2. Four women were diagnosed with GDM at 12 weeks of pregnancy and the other eight at 24 weeks of pregnancy. In general, micronutrient intake and status levels were not significantly associated with glucose homeostasis markers over the course of the pregnancy (Table 3). However, total iron intake was inversely associated with fasting glucose levels (β = −0.069 mmol/L for each FE µg (*p* = 0.013) and HbA1c levels (β = −0.4843 mmol/L for each FE µg, *p* = 0.002). A higher diet quality was associated with lower fasting glucose (β = −0.006 mmol/L for each DHD15-index point, *p* = 0.017).

## 4. Discussion

In this study, folate, vitamin B6, vitamin B12, vitamin D and iron intake significantly changed from preconception to 24 weeks of pregnancy, which was predominantly attributable to changes in supplemental intake. Moreover, intakes of folate, vitamin B6 and vitamin D were significantly positively associated with their status markers. Total iron intake and diet quality were inversely associated with fasting glucose levels, while iron was additionally inversely associated with HbA1c levels. Other micronutrient intakes and status markers were not associated with glucose homeostasis. 

Our participants increased their energy intake with on average 7% from preconception to 24 weeks of pregnancy. At the same time, no changes in macronutrient composition, dietary folate, vitamin B6, vitamin B12, vitamin D and iron intake, and diet quality were observed. These findings are in line with other studies investigating changes in dietary intake during pregnancy [37,38]. Recent studies also indicate that many women enter pregnancy with suboptimal micronutrient intakes [3,39,40]. As adequate micronutrient stores are essential to ensure an optimal intrauterine environment for fetal development [41], our data and already published research suggest an important window of opportunity to improve diet quality [42,43,44]. 

Supplementation has been shown to be effective in improving micronutrient status and meeting recommendations. An adequate folate status is known to be of major importance during the first trimester of pregnancy. Consequently, pregnant women globally are advised to use a folic acid supplement starting at least 4 weeks before conception. Accordingly, 89% of the women in this study population used at least one supplement at 12 weeks of pregnancy, which is comparable with percentages reported in other studies [39,45,46]. The most commonly used supplements were folic acid, folic acid combined with vitamin D and prenatal multivitamin supplements. The usage, number and type of supplements changed throughout pregnancy, with the most pronounced being the doubling of supplemental folate intake (mean 680 FE μg) at 12 weeks of pregnancy compared to the mean an intake of 340 FE μg at preconception and at 24 weeks of pregnancy. The drop in supplemental folate intake from 12 to 24 weeks of pregnancy has also been observed in other studies [39,45]. This drop is most likely related to the recommendation to take folic acid supplements until the 12th week of gestation [47]. In contrast to the known beneficial effects of folate supplementation, supplementation may also result in excess intake above the upper level of intake that might negatively affect health of the offspring. In our population, 29 women (27%) consumed folate above the upper level of intake (1000 FE µg). For other micronutrients, we observed no intake above upper levels. As a recent study has found a link between high doses of folic acid during pregnancy (>5 mg/day) and impaired psychomotor development at 12–23 months of age, folate intake above the upper level is of concern [48]. Although the maximum folate intake in our population was 1.7 mg/day, such data indicate that excess nutrient intake should not be taken too lightly, and that more research is needed to unravel the adverse effects of excess micronutrient intake, especially for potential adverse effects in the offspring.

We observed significant intake-status associations for folate, vitamin D and vitamin B6, but not for vitamin B12 and iron. This is in line with findings from validation studies in pregnant populations using nutritional biomarkers as reference instrument to validate dietary intake assessment methods [49]. Supplemental folate intake was the strongest predictor for folate status levels. For other micronutrients, intake could not (entirely) explain observed changes in status levels. In general, we observed that the status markers of water-soluble B-vitamins decreased from the end of the first trimester to the end of the second trimester. This decrease was likely attributable to haemodilution, the increase in blood volume in the second and third trimester [22,50], in addition to changes in micronutrient intake. The absence of an intake-status association for vitamin B12 might be due to a small range of intake, which limits the ability to detect an association. Another explanation could be the fact that average intake was >4 μg/day whereas recommended dietary intake is 2.4 μg/day and a so called saturation of intake has occurred. The absence of an association between iron intake and ferritin levels may be explained by the fact that gestational ferritin levels are affected by haemodilution and increased erythropoiesis [51]. 

In our study, vitamin D status, i.e., 25(OH)D, levels increased from preconception to 24 weeks of pregnancy. This is in line with findings of most [25,52,53], but not all [54], other studies investigating vitamin D levels over the course of the pregnancy. This observed increase in 25(OH)D in our study remained after adjustments for dietary and supplemental intake, and other known potential confounders such as season, BMI and weight change. A possible explanation for the observed increase in 25(OH)D levels is increased production, potentially driven by placental vitamin D metabolism [55]. Vitamin D is essential for bone development, has important immune functions [56], and deficiencies of this nutrient have been linked to adverse pregnancy-outcomes including preeclampsia, low birthweight, neonatal hypocalcaemia, poor postnatal growth and bone fragility [57]. Therefore, there has been increasing attention for the high prevalence of vitamin D deficiency in general and during pregnancy, with prevalences reported of up to 84%, depending on the country of residence [57]. However, it needs to be emphasized that the applied cut-offs for vitamin D deficiency are based on cut-offs that have been set for the general population [58]. Since we and others [25,52,53] observed that 25(OH)D levels increase throughout pregnancy, the usability of these general criteria for vitamin D deficiency in pregnant population might depend on gestational age. 

Although the evidence regarding a beneficial effect of a healthy diet in GDM prevention is growing, research regarding the intake and status of micronutrients and their association with GDM is limited. We observed a significant inverse association between diet quality and fasting glucose levels, and significant inverse associates between iron intake and fasting glucose and HbA1c levels, but not for the other micronutrients. In contrast to our results, two cross-sectional Asian studies reported that higher folate levels were associated with a higher risk of GDM, whereas vitamin B12 status was inversely associated with GDM risk [18,19]. A retrospective study in the UK also observed a lower vitamin B12 status in women with GDM, but did not find an association of folate status with GDM risk [20]. Furthermore, several observational studies found an inverse association of vitamin D status with GDM development, but this could not be confirmed in randomized controlled trials [17]. In agreement, we also did not observe a significant association between vitamin D intake, 25(OH)D status levels and markers of glucose homeostasis. 

We observed inverse associations of iron intake with fasting glucose and HbA1c levels, and null associations for ferritin and glucose homeostasis markers. These findings are in contrast with a recent review that concluded that there is a potential link between higher iron status and increased risk of GDM [16]. Mechanistically, adequate iron stores are critical for normal beta cell function and glucose homeostasis, whereas excess iron may disrupt glucose homeostasis, by damaging beta cells function, increasing oxidative stress and impaired insulin signaling [59,60,61]. Existing literature suggests that the association between higher dietary intake and higher risk of GDM is most pronounced for dietary heme iron and serum ferritin, but mixed results were observed for total iron and supplementary iron [16]. Unfortunately, we were not able to distinguish between heme and non-heme iron intake. Moreover, we did not find an association between serum ferritin and glucose homeostasis markers, which may be related to the limited sample size. As iron supplementation is often prescribed to prevent or treat iron deficiency anaemia, the potential association of a higher GDM risk with higher iron status warrants more research. 

There is ample evidence of the beneficial role that diet might play in prevention of GDM [8,9,10]. Our observation of an inverse association between diet quality and fasting glucose is in line with these studies. However, it is notable that we did not observe a significant association of diet quality with 2-h glucose levels. Research on type-2 diabetes, which has similarities in disease aetiology with GDM, has shown that lifestyle factors such as unhealthy diet, physical inactivity and smoking are associated with higher 2-h glucose levels, but not with fasting plasma glucose [62], which is in contrast to our findings. More research on the association between fasting and 2-h glucose levels with diet quality in women with GDM is needed to confirm our observation, and consequently, explore possible explanations. Furthermore, it should be noted that the effect size of our association, −0.006 mmol/L per DHD15 point, might not be clinically relevant. With a range of 0–150 points a maximum theoretical benefit of −0.9 mmol/L could only be achieved by someone going from non-adherence to complete adherence to the Dutch dietary guidelines. 

The major strengths of the present study include its prospective design, inclusion of measurements before conception and detailed information on dietary intake, supplemental intake and micronutrient status markers. However, the present study also has limitations. Firstly, due to the extensive nature of our study, with measurements at preconception as well as during pregnancy, including a visit to the research center, an OGTT and various dietary assessment at each time point, our sample size was limited, making our study potentially underpowered to assess significant associations. We did not correct for multiple testing, and as such, we cannot rule out that some observed significant associations occurred by chance. However, we were able to use continuous effect measures, and had extensive information regarding dietary intake, supplement intake, serum levels and potential confounders. Secondly, due to various reasons (women who did not get pregnant, sickness, holidays etc.); almost half of the participants completed only one measurement. Thirdly, participants included in the present study were mainly highly educated, with oversampling of women with a high risk of GDM. These factors may limit external validity to the general population of pregnant women. Fourthly, although the FFQ was validated in women of reproductive age, it was not specifically validated for use in pregnant women. Furthermore, the DHD15-index was designed to assess diet quality based upon dietary guidelines for the Dutch general population [32]. These guidelines apply also for pregnant women, with the addition to the use of folic acid supplements at least 4 weeks prior to conception until at least 12 weeks of conception [63]. This additional guideline for pregnant women is not included in the DHD15-index. Lastly, as the primary outcome of the study was the development of GDM, the last measurement was at 24 weeks of pregnancy. To accurately describe changes in micronutrient levels over the pregnancy additional measurements after 24 weeks of pregnancy would give a more complete picture. 

## 5. Conclusions

In this study, the intake and status levels of micronutrients vary throughout pregnancy, which was predominantly related to variation in supplemental intake and not dietary micronutrient intake. Diet quality was inversely associated with fasting glucose levels and iron intake was inversely associated with fasting glucose and HbA1c levels, whereas vitamin D, B6, B12 and folate were not associated with markers of glucose homeostasis. Larger studies need to confirm our results, and more research is needed with respect to the role of micronutrients in relation to GDM development.

## Figures and Tables

**Table 1:** Characteristics of the study population according to measurement moment: preconception (T0), 12 weeks gestation (T1) and 24 weeks gestation (T2).

	Total N = 105	T0 N = 67	T1 N = 53	T2 N = 66	*p*-Value ^a^
Gestational age	-	−13 (−25; −1)	13 (12.3; 14.9)	25 (24; 26)	<0.001
Age (years)	31.9 (29.8; 34.3)	31.7 (29.0; 34.1)	31.9 (29.7; 33.8)	32.6 (30.7; 34.7)	0.840
Educational level (%)					0.940
Low	4.7	4.5	5.7	6.1	
Moderate	34.3	32.8	26.4	31.8	
High	61.0	62.7	67.9	62.1	
Western ethnicity (%)	94.3	95.5	98.1	95.5	0.697
Smokers (%)	9.5	11.9	1.9	1.5	0.012
Parity (% ≥1 child)	92.4	91.0	96.2	94.0	0.511
Nausea during pregnancy (%)	-	-	90.0	53.9	-
Vomitting during pregnancy (%)	-	-	32.0	20.0	-
BMI (kg/m^2^)	25.0 (22.6; 28.9)	24.4 (22.2; 28.4)	24.7 (22.5; 29.2)	27.0 (24.6; 31.4)	0.007
Waist circumference (cm)	88.5 (79.0; 95.5)	84.8 (78.0; 92.5)	88.4 (80.8; 96.4)	98.5 (90.5; 106.0)	<0.001
Hip circumference (cm)	105.5 (100.0; 113.0)	105.3 (98.5; 112.0)	105.9 (99.0; 113.8)	108.4 (101.4; 116.8)	0.135
Physical activity (MET min/week)	1200 (750; 1860)	1320 (870; 2029)	853 (671; 1358)	978 (390; 1459)	0.0234
Energy intake (kJ)	8583 (6885; 9623)	8583 (6713; 9462)	8465 (7009; 9975)	9189 (7432; 10541)	0.133
Carbohydrates (E%)	46.5 (43.2; 49.7)	45.4 (42.3; 48.6)	46.5 (45.2; 50.3)	48.1 (44.8; 50.3)	0.030
Fat (E%)	35.4 (32.4; 37.7)	36.2 (32.5; 38.8)	35.2 (32.7; 37.5)	34.1 (31.8; 38.2)	0.343
Protein (E%)	15.1 (13.8; 16.7)	15.6 (14.3; 16.9)	14.9 (13.7; 16.6)	14.5 (13.3; 15.8)	0.045
Alcohol (%)					<0.001
Abstainers	80.1	55.2	96.2	92.4	
0–1 standard glass/day	17.7	38.8	3.8	7.6	
>1 stanard glass/day	2.2	6.0	0.0	0.0	
Blood sampling between December and April (%)	23.1	17.9	26.4	25.8	0.448

Values are median (25th percentile; 75th percentile) or percentage; ^a^ *p*-values from χ^2^ for categorical values or ANOVA for continuous variables for differences between T0, T1, and T2.

**Table 2:** Regression coefficients (β) of association of pregnancy (time), dietary intake and supplemental intake with changes in folate, vitamin B6, vitamin B12, 25(OH)D and ferritin blood levels.

Outcome	Characteristic	β	95% CI	*p*-Value
Folate status ^b^ (nmol/l)	Time—12 weeks pregnancy ^a^	3.14	−1.33; 7.62	0.166
	Time—24 weeks pregnancy ^a^	0.20	−3.69; 4.10	0.917
	Supplemental folate intake (FE μg)	0.026	0.020; 0.031	<0.001
	Dietary folate intake (FE μg)	−0.015	−0.044; 0.014	0.297
Vitamin B6 status ^c^ (nmol/L)	Time—12 weeks pregnancy ^a^	−1.70	−9.10; 5.70	0.647
	Time—24 weeks pregnancy ^a^	−10.3	−17.5; −3.25	0.005
	Supplemental vitamin B6 intake (mg)	2.15	0.89; 3.43	0.001
	Dietary vitamin B6 intake (mg)	1.17	−9.42; 11.8	0.826
Vitamin B12 status ^d^ (pmol/L)	Time—12 weeks pregnancy ^a^	−55.2	−74.0; −36.4	<0.001
	Time—24 weeks pregnancy ^a^	−91.1	−109; −73.1	<0.001
	Supplemental vitamin B12 intake (μg)	0.52	−0.55; 1.59	0.336
	Dietary vitamin B12 intake (μg)	5.06	−1.76; 11.9	0.143
25(OH)D status ^e^ (nmol/L)	Time—12 weeks pregnancy ^a^	17.0	9.90; 24.0	<0.001
	Time—24 weeks pregnancy ^a^	33.5	25.7; 41.3	<0.001
	Supplemental vitamin D intake (μg)	1.03	0.51; 1.54	<0.001
	Dietary vitamin D intake (μg)	6.66	3.78; 9.55	<0.001
Ferritin status ^f^ (µg/L)	Time—12 weeks pregnancy ^a^	−5.75	−12.4; 0.88	0.088
	Time—24 weeks pregnancy ^a^	−23.0	−28.2; −17.8	<0.001
	Supplemental iron intake (mg)	0.12	−0.12; 0.36	0.328
	Dietary iron intake (mg)	−0.22	−1.45; 1.01	0.723

^a^ Preconception is reference category. ^b^ Estimates are adjusted for age, education level, season of blood collection, BMI, energy intake, vitamin B12 and B6 intake. ^c^ Estimates are adjusted for age, educational level, vitamin B12 intake and energy intake. ^d^ Estimates are adjusted for parity, season of blood collection, BMI, energy intake, vitamin B6 intake. ^e^ Estimates are adjusted for education level, parity, season of blood collection, BMI and energy intake. ^f^ Estimates are adjusted for alcohol intake, nausea during pregnancy, BMI and energy intake.

**Table 3:** Regression coefficients (β) of associations between pregnancy (model 1), diet quality as assessed with DHD15-index (model 2), micronutrient intake (model 3) and micronutrient status (model 4) and fasting glucose, 2 h glucose levels and HbA1c.

Outcome	Model	Exposure Variable	β	95% CI	*p*-Value
Fasting glucose	(1) ^b^	Time—12 weeks pregnancy ^a^	−0.252	−0.386; −0.117	0.001
		Time—24 weeks pregnancy ^a^	−0.425	−0.570; −0.281	<0.001
	(2) ^c^	DHD-15 index score	−0.006	−0.011; −0.001	0.017
	(3) ^c^	Total folate intake (FE μg)	0.000	−0.001; 0.001	0.929
		Total vitamin B6 intake (mg)	−0.005	−0.064; 0.055	0.869
		Total vitamin B12 intake (μg)	−0.014	−0.221; 0.193	0.893
		Total vitamin D intake (μg)	0.013	−0.037; 0.062	0.612
		Total iron intake (mg)	−0.069	−0.124; 0.015	0.013
	(4) ^d^	Serum folate	−0.003	−0.007; 0.002	0.261
		Serum 25(OH)D	0.001	−0.001; 0.003	0.310
		Whole blood vitamin B6	0.000	0.000; 0.000	0.843
		Serum vitamin B12	0.000	−0.001; 0.001	0.960
		Serum ferritin	0.000	−0.004; 0.004	0.966
2 h glucose	(1) ^b^	Time—12 weeks pregnancy ^a^	0.375	0.041; 0.71	0.029
		Time—24 weeks pregnancy ^a^	1.079	0.653; 1.505	<0.001
	(2) ^c^	DHD-15 index score	−0.011	−0.025; 0.002	0.100
	(3) ^c^	Total folate intake (FE μg)	−0.001	−0.004; 0.002	0.590
		Total vitamin B6 intake (mg)	−0.046	−0.209; 0.118	0.580
		Total vitamin B12 intake (μg)	0.121	−0.444; 0.686	0.671
		Total vitamin D intake (μg)	0.030	−0.094; 0.154	0.635
		Total iron intake (mg)	−0.064	−0.226; 0.097	0.429
	(4) ^d^	Serum folate	−0.002	−0.013; 0.008	0.656
		Serum 25(OH)D	0.000	−0.007; 0.007	0.996
		Whole blood vitamin B6	−0.001	−0.003; 0.002	0.660
		Serum vitamin B12	0.000	−0.002; 0.002	0.788
		Serum ferritin	0.005	−0.005; 0.014	0.325
HbA1c	(1) ^b^	Time—12 weeks pregnancy ^a^	−1.963	−2.571; −1.355	<0.001
		Time—24 weeks pregnancy ^a^	−3.360	−4.004; −2.716	<0.001
	(2) ^c^	DHD-15 index score	−0.016	−0.044; 0.012	0.248
	(3) ^c^	Total folate intake (FE μg)	0.001	−0.006; 0.007	0.847
		Total vitamin B6 intake (mg)	−0.167	−0.478; 0.144	0.288
		Total vitamin B12 intake (μg)	−0.138	−1.231; 0.954	0.801
		Total vitamin D intake (μg)	0.087	−0.172; 0.346	0.505
		Total iron intake (mg)	−0.484	−0.783; −0.184	0.002
	(4) ^d^	Serum folate	0.021	−0.001; 0.044	0.066
		Serum 25(OH)D	−0.006	−0.018; 0.007	0.364
		Whole blood vitamin B6	−0.008	−0.023; 0.006	0.249
		Serum vitamin B12	0.002	−0.003; 0.006	0.506
		Serum ferritin	−0.011	−0.032; 0.010	0.288

^a^ Preconception is reference category. ^b^ Adjusted for age, ethnicity, education level, parity, history of GDM, BMI; ^c^ Adjusted for time, age, ethnicity, parity, history of GDM, BMI, energy intake; ^d^ Adjusted for time, age, ethnicity, parity, history of GDM, BMI.

## Supplementary Materials

The following are available online at https://www.mdpi.com/2072-6643/11/2/460/s1, Table S1: Micronutrient intake, micronutrient status, diet quality and glucose homeostasis markers of the study population according to measurement moment: preconception (T0), 12 weeks gestation (T1) and 24 weeks gestation (T2).
