# Supplementary material for: Changes in Micronutrient Intake and Status, Diet Quality and Glucose Tolerance from Preconception to the Second Trimester of Pregnancy
Source: Nutrients. 2019 Feb 22;11(2):460. doi: 10.3390/nu11020460 (PMC6412670; doi:10.3390/nu11020460)
Supplement: Supplementary file 1 [file nutrients-11-00460-s001.pdf]

Supplemental table S1. Micronutrient intake, micronutrient status, diet quality and glucose homeostasis markers of the study population according to measurement moment: preconception (T0), 12 weeks gestation (T1) and 24 weeks gestation (T2).

	T0<br>N=67	T1<br>N=53	T2<br>N=66	P-value*<br>T0 vs T1	P-value<br>T0 vs T2	P-value<br>T1 vs T2
DHD15 score	80.7 (1.6)	79.2 (1.8)	78.0 (1.6)	0.442	0.159	0.417
Plasma folate (nmol/L)	29.3 (2.2)	41.1 (2.1)	29.7 (2.1)	<0.001	0.8830	<0.001
Total folate intake (FE µg/d)	642.5 (46.5)	905.2 (49.9)	680.8 (47.8)	<0.001	0.517	<0.001
- Dietary intake	281.5 (10.6)	283.7 (9.5)	285.8 (10.7)	0.830	0.712	0.773
- Supplemental intake	362.4 (45.1)	624.8 (47.6)	395.6 (44.4)	<0.001	0.561	<0.001
Whole blood vitamin B6 (nmol/L)	89.8 (3.4)	88.7 (2.9)	80.0 (2.8)	0.765	0.012	0.008
Total vitamin B6 intake (mg/d)	3.0 (0.5)	3.7 (0.5)	3.3 (0.4)	0.298	0.634	0.025
- Dietary intake	1.7 (0.1)	1.8 (0.1)	1.8 (0.1)	0.137	0.047	0.137
- Supplemental intake	1.3 (0.4)	2.0 (0.6)	1.4 (0.4)	0.319	0.875	0.009
Serum vitamin B12 (pmol/L)	308.4 (10.8)	258.3 (11.0)	210.3 (7.6)	<0.001	<0.001	<0.001
Total vitamin B12 intake (µg/d)	8.8 (2.4)	6.7 (0.7)	6.6 (0.7)	0.374	0.354	0.682
- Dietary intake	4.3 (0.2)	4.3 (0.2)	4.4 (0.2)	0.840	0.617	0.431
- Supplemental intake	4.4 (2.4)	2.5 (0.7)	2.2 (0.6)	0.432	0.354	0.239
Serum 25(OH)D (nmol/L)**	62.1 (3.0)	77.4 (3.1)	88.5 (4.2)	<0.001	<0.001	0.001
Total vitamin D intake (µg/d)	7.7 (0.8)	10.4 (0.7)	8.9 (0.6)	0.002	0.225	0.064
- Dietary intake	3.5 (0.2)	3.3 (0.2)	3.3 (0.2)	0.408	0.469	0.978
- Supplemental intake	4.1 (0.8)	7.1 (0.7)	5.6 (0.6)	<0.001	0.141	0.035
Plasma ferritin (µg/L)	31.7 (2.2)	31.4 (2.5)	12.8 (1.2)	0.900	<0.001	<0.001
Total iron intake (mg/d)	14.1 (0.9)	19.7 (1.0)	19.4 (1.1)	<0.001	<0.001	0.806
- Dietary intake	10.7 (0.3)	10.5 (0.3)	10.9 (0.3)	0.535	0.599	0.109
- Supplemental intake	3.5 (0.8)	9.2 (1.0)	8.5 (0.9)	<0.001	<0.001	0.495

Values are mean (SEM).

* P value obtained using LS means to test for difference between measurement moments.

** Adjusted for season.
